# Flexoelectricity Driven Fano Resonance in Slotted Carbon Nanotubes for Decoupled Multifunctional Sensing

**DOI:** 10.34133/2021/9821905

**Published:** 2021-12-29

**Authors:** Jinlong Ren, Yingchao Liu, Xingqiang Shi, Guangcun Shan, Mingming Tang, Chaocheng Kaun, Kunpeng Dou

**Affiliations:** ^1^College of Information Science and Engineering, Ocean University of China, Qingdao 266100, China; ^2^Key Laboratory of Optic-Electronic Information and Materials of Hebei Province, Institute of Life Science and Green Development, College of Physics Science and Technology, Hebei University, Baoding 071002, China; ^3^Institute of Precision Instrument and Quantum Sensing, School of Instrumentation Science and Opto-Electronics Engineering, Beihang University, Beijing 100191, China; ^4^Institute of Experimental Physics, Saarland University, 66123 Saarbrücken, Germany; ^5^School of Geosciences, China University of Petroleum, Qingdao 266580, China; ^6^Research Center for Applied Sciences, Academia Sinica, Taipei 11529, China

## Abstract

Multifunctionality, interference-free signal readout, and quantum effect are important considerations for flexible sensors equipped within a single unit towards further miniaturization. To address these criteria, we present the slotted carbon nanotube (CNT) junction features tunable Fano resonance driven by flexoelectricity, which could serve as an ideal multimodal sensory receptor. Based on extensive ab initio calculations, we find that the effective Fano factor can be used as a temperature-insensitive extrinsic variable for sensing the bending strain, and the Seebeck coefficient can be used as a strain-insensitive intrinsic variable for detecting temperature. Thus, this dual-parameter permits simultaneous sensing of temperature and strain without signal interference. We further demonstrate the applicability of this slotted junction to ultrasensitive chemical sensing which enables precise determination of donor-type, acceptor-type, and inert molecules. This is due to the enhancement or counterbalance between flexoelectric and chemical gating. Flexoelectric gating would preserve the electron–hole symmetry of the slotted junction whereas chemical gating would break it. As a proof-of-concept demonstration, the slotted CNT junction provides an excellent quantum platform for the development of multistimuli sensation in artificial intelligence at the molecular scale.

## 1. Introduction

Mechanically flexible electronic devices offer great potential for applications in health monitoring, robotics, and prosthetics, which are expected to play a crucial role in continuously collecting data from the human body to capture meaningful health status changes in time for preventive intervention.

One consideration for this human-machine interface is device multifunctionality to fulfill simultaneous multistimulus sensing [1]. To replicate such skin-like capabilities, previous efforts have been put through the integration of different types of sensors in the stacked form by lamination [2–4]. However, distinct interconnections are needed to link each sensor layer to relay signals to external data acquisition instrument, and this increases the structure complexity [2]. In addition, the stacked configuration (multilayered layout) would reduce the mechanical responses to external deformations due to the mechanical interference across different layers, which is a core challenge in achieving high performance with a multimodal sensor [2].

To decouple the signal interference, more emphasis is put on achieving multimodality within a single sensory unit. One alternative approach is to exploit heterogeneous sensing materials. For example, Tien et al. developed a field-effect transistor (FET) sensor platform with piezo-pyroelectric nanocomposite as gate dielectrics and piezo-thermoresistive organic semiconductor as channel [5]. The two subcomponents respond to strain and temperature disproportionally. Zhang et al. employed porous polyurethane as a supporting microstructure frame and organic thermoelectric material to achieve a dual-parameter sensor. The independent piezoresistive and thermoelectric effects in this single device enable simultaneous monitoring of strain and temperature [6]. However, the integration of heterogeneous sensing materials may arouse the structural complexity [5]. To simplify the structural integration, You et al. report advances of multimodal sensor using only electrolyte as the working material [7]. The charge relaxation time (intrinsic electrical parameter) and the normalized capacitance (extrinsic electrical parameter) of this ionic conducting material are strain-independent and temperature-insensitive, which are the naturally decoupled signal readout for thermal and mechanical perception, respectively.

On the other hand, miniaturization of integrated circuits has been a long-lasting pursuit for electronic devices in the past few decades. In spite of these abovementioned attempts done in terms of multimodal sensory systems at the microscale, it becomes increasingly important to explore the quantum effect in the nanoscale (or molecular scale) when approaching the limit of Moore's law [8].

**Figure 1:**
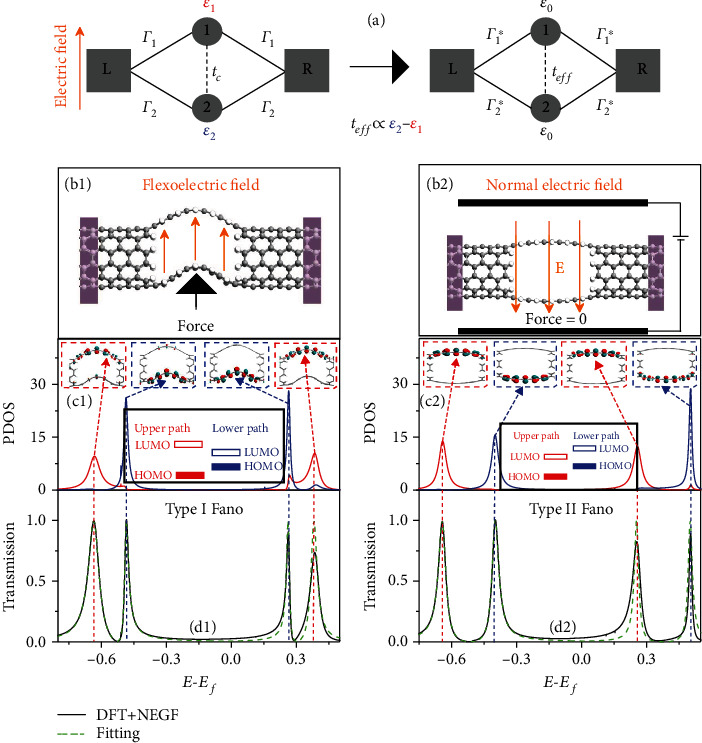
(a) and (b) Illustrative schematic model of parallel quantum dots realized by a slotted CNT junction. (c1)–(d2) Different Fano resonances driven by flexoelectric and normal electric fields. Projected density of states (PDOS) (c1) and (c2) and transmission spectra (d1) and (d2) for the two types of Fano resonances. In (d1) and (d2), the Fano model (dashed green lines) in (a) can accurately account for the DFT + NEGF results (solid black lines). The inserts in (c1) and (c2) are the Bloch wavefunctions of frontier orbitals for upper- and lower-paths in the slotted CNT junction.

To circumvent this cumbersome issue of multifunctional sensing towards further miniaturization, biomimicry has been a long-lasting inspiration for the novel yet simple designs and technological innovations. Herein, we describe how the parallel slits in the spider's legs served as the inspiration for developing a simultaneously multistimuli responsive unit. Notably, we introduce the effective parallel slit architecture into carbon nanotube (CNT) with tunable quantum interference effect for integrating the multifunctional flexible electronics with high performance to satisfy the next-generation intelligent sensor. Such architecture can integrate low-dimensional materials to enable functional transformation from the deformation to changes in their physical, mechanical, electric, and chemical properties. To go beyond the existing transduction strategies, feedback functions from Fano resonance [9] driven by flexoelectricity are employed to carry and transduce information from external stimuli. There are two representative examples to achieve Fano phenomenon in dominating electron transport in nanoscale junctions: (1) a pendant unit connected to a main path and (2) multiple paths under external electrostatic or magnetic field [10]. Both lead to asymmetric transmission profiles, and the asymmetry parameter is described as Fano factor *t*. Benefiting from the latter example, we show that the effective Fano factor in parallel paths (*t*_*eff*_, energy splitting between the two paths, compare Figure 1(a) and equation (1)) can be used as a temperature-insensitive extrinsic variable for sensing the strain, and the Seebeck coefficient can be used as a strain-insensitive intrinsic variable for detecting temperature; thus, decoupling the two signals. We further demonstrate the applicability of this concept platform to ultrasensitive chemical sensing for three types of representative molecules—donor-like, acceptor-like, and inert molecules. Good determinations of single molecule are available by the values and the sign of relative changes in effective Fano factor due to the enhancement or counterbalance between flexoelectric and chemical gating.

## 2. Results

The slit geometry of the spider's sensory organs has been successfully exploited in a Pt film for attaining ultrahigh mechanosensitivity [11]. Due to the varied spacing in the crack-shaped slit on the metal film by pressure or vibration, measured electrical conductance across the cracks experienced on and off states. Not all the cracking edges are in contact with each other and there exists even about 5 nm crack spacing in the Pt strip even without strain. Quantum effect is thought to play a tiny role in addressing the crosstalk between the opposite edges of the crack during their cyclical disconnection–reconnection process and hence the sensor mechanism [12].

Here, we mimic the parallel arrangement of slit geometry to design multifunctional sensors based on CNT junctions, where the sensory performance is dominated by quantum interference effect, namely, Fano resonances [9]. This quantum phenomenon is related with the crosstalk between the parallel paths [13] under flexoelectric field due to bending deformation. To capture the essential physics, we choose the parallel quantum dot systems under the external electric field as the starting point (cf.  Figure 1(a)). Both paths are assumed to be symmetrically coupled to the left and right electrodes via the coupling constants Γ_1_ or Γ_2_ (Figure 1(a)). The crosstalk between the parallel paths through the direct tunneling *t*_*c*_ is negligible and after the model transformation (Figure 1(a)), the new term *t*_*eff*_ boosted by the electric field can be used to describe the effective coupling strength between the two paths which are still symmetrically coupled to the left and right electrodes via the coupling constants Γ_1_^∗^ or Γ_2_^∗^. According to equation 20 in Ref. [13] with zero magnetic field, the transmission spectrum for our artificial Fano system can be expressed as

(1) $$\begin{array}{c} T\left(E\right)=\frac{{\left(\Gamma_{1}^{*}+\Gamma_{2}^{*}\right)}^{2}{\left[E-\varepsilon_{0}-\left[2\sqrt{\Gamma_{1}^{*}\Gamma_{2}^{*}}/\left(\Gamma_{1}^{*}+\Gamma_{2}^{*}\right)\right]t_{eff}\right]}^{2}}{\left\{{\left[E-\left(\varepsilon_{0}+t_{eff}\right)\right]}^{2}+{\left[{\left(\sqrt{\Gamma_{1}^{*}}-\sqrt{\Gamma_{2}^{*}}\right)}^{2}/2\right]}^{2}\right\}\left\{{\left[E-\left(\varepsilon_{0}-t_{eff}\right)\right]}^{2}+{\left[{\left(\sqrt{\Gamma_{1}^{*}}+\sqrt{\Gamma_{2}^{*}}\right)}^{2}/2\right]}^{2}\right\}}, \end{array}$$

where *ε*_0_ = (*ε*_1_ + *ε*_2_)/2 and $t_{eff}=\sqrt{{\left(\varepsilon_{1}-\varepsilon_{2}\right)}^{2}/4+t_{c}^{2}}\approx \left|\varepsilon_{1}-\varepsilon_{2}\right|/2$ can be approximated as the energy splitting between upper and lower paths under electric field; *ε*_1_ (*ε*_2_) denotes the highest occupied molecular orbital, HOMO or the lowest unoccupied molecular orbital, LUMO of upper (lower) path, the degeneracy of *ε*_1_ and *ε*_2_ will be broken upon external electric fields. Note that *t*_*eff*_ is proportional to the energy difference between the two paths, which is the energy splitting in HOMO (or LUMO) between the two paths in our systems. The details of conversion from equation 20 in Ref. [13] to the simplified equation (1) can be found in Supplementary Materials.

**Figure 2:**
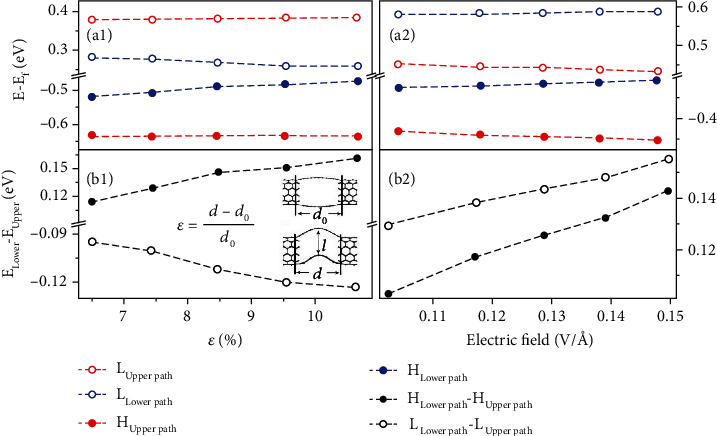
Evolution of frontier orbitals for upper and lower paths as a function of strain deformation (a1) and the normal electric field (a2). Evolution of the energy slitting between frontier orbitals of upper and lower paths as a function of strain deformation (b1) and the normal electric field (b2). The definition of strain is shown in the insert of (b1), based on the lengths of the nanocracked paths without and with bending. The vertical separation between the upper-to-lower paths is denoted with *l*. “H” and “L” in this figure mean HOMO and LUMO, respectively.

Then, we perform a comparative analysis to clarify the origin of the Fano resonance in a slotted CNT junction under the externally flexoelectric and normal electric fields as depicted in Figures 1(b1)–1(d2). The normal electric field is applied to the flat CNT junction without strain (Figure 1(b2)). The projected density of states (PDOS) in Figures 1(c1) and 1(c2) indicate that both kinds of fields will break the energy degeneracy of frontier orbitals (HOMOand LUMO) in the two paths but in different manners (the width of each path is equivalent to two zigzag chains). The energy alignment in the former case displayed in Figure 1(c1) is reminiscent of straddling type I band alignment in semiconducting heterostructures, whereas the latter case provided in Figure 1(c2) shares the staggered character of type II band alignment. Thus, one can define the Fano resonances in the slotted CNT junctions as type I and type II originated from flexoelectric field and normal external electric field, respectively. The density functional theory (DFT) and nonequilibrium Green's function (NEGF) transmission results shown in Figures 1(d1) and 1(d2) are well reproduced by fitting equation (1), which confirm the model analysis in Figure 1(a). We further probed the evolution of frontier orbitals in upper and lower paths as a function of strain degree or the strength of normal electric field (cf. Figures 2(a1) and 2(a2)). Both preserve the type I or type II band alignment in the considered ranges, and we extract the evolution of the frontier orbital energy slitting between the two paths (presented in Figures 2(b1) and 2(b2)) which also represents the effective coupling strength, *t*_*eff*_ ∝ |*E*_upper_ − *E*_lower_|. It should be noted that the nearly linear variation of energy slitting or *t*_*eff*_ with ascending strain endows this slotted CNT junction applicable as mechanically-tunable electronic components. Compared with previously explored sensors in detection of strain, the signal readout generally adopts current or resistance response to strain deformation [1]. Here, we employ a novel feedback function (*t*_*eff*_) beyond the existing transduction strategies.

**Figure 3:**
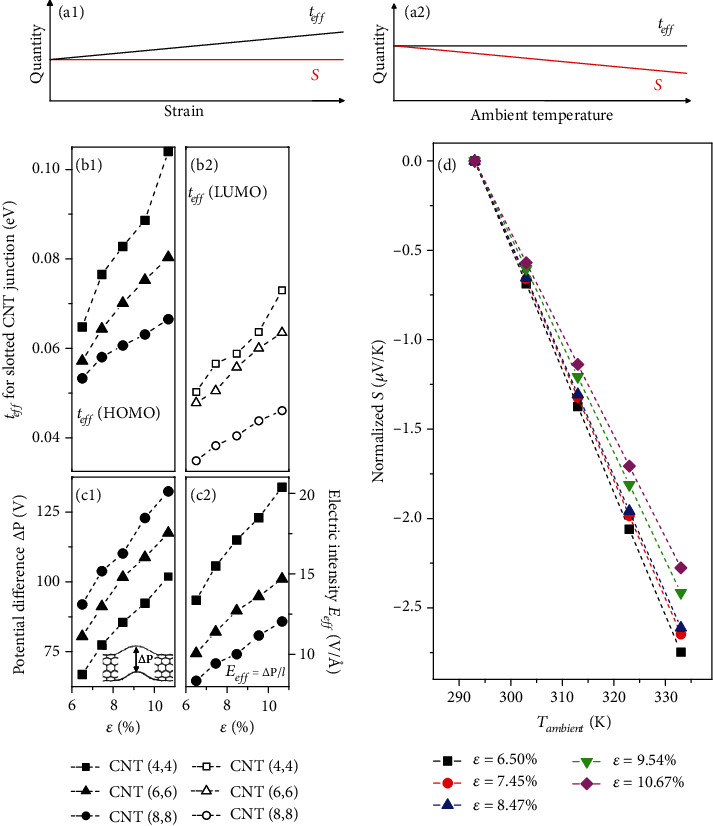
Conceptual plots of the strain-insensitive intrinsic Seebeck coefficient *S* (a1) and the temperature-insensitive extrinsic variable *t*_*eff*_ (a2). (b1) and (b2) The effective Fano factor *t*_*eff*_ of HOMO and LUMO for three sets of bendable CNT junctions. (c1) and (c2) Potential difference (△*P*) and corresponding estimated electric intensity (*E*_*eff*_) between upper and lower paths for three sets of bendable CNT junctions. The upper-to-lower path separation, *l*, is labeled in the inset of Figure 2(b1). (d) Normalized Seebeck coefficient *S* of the bendable slotted CNT (6,6) junction at strain ranging from 6.50% to 10.67%.

We proceed to demonstrate that the slotted CNT junction could detect different external stimuli simultaneously and relay independent signals for evaluating strain and temperature, as illustrated in Figures 3(a1) and 3(a2). The thermo-mechanical decoupling is realized by Seebeck coefficient (*S*) and *t*_*eff*_. These two variables prevent cross-sensitivity.

### 2.1. For the *t*_*eff*_

We scrutinize the *t*_*eff*_ response of CNT junctions with three different radii to strain deformation, as plotted in Figures 3(b1) and 3(b2). As the junction experienced bending strain ranging from 6.50% to 10.67%, the *t*_*eff*_ (proportional to the energy splitting in HOMO or LUMO in the two paths) scale almost linearly with strain changes. The evolution trend of electric intensity (*E*_*eff*_) between upper and lower paths of CNT junctions with strain is qualitatively the same with that for both the HOMO and the LUMO related *t*_*eff*_. Thus, this reflects bending induced flexoelectricity does play a role in affecting the energy splitting of frontier orbitals between the two paths.

### 2.2. For the Seebeck Coefficient

The Seebeck coefficient (*S*) of this molecular junction is a key metric for evaluating the thermal response of temperature sensing and is defined as [14]

(2) $$\begin{array}{c} S=-\frac{\pi^{2}k_{B}^{2}T_{ambient}}{3e}{\left.\frac{\partial \ln\left[T\left(E\right)\right]}{\partial E}\right|}_{E=E_{F}}=-2.445\times 10^{-2}\times T_{ambient}{\left.\frac{\partial \ln\left[T\left(E\right)\right]}{\partial E}\right|}_{E=E_{F}}, \end{array}$$

where *k*_*B*_ is the Boltzmann constant and *E*_*F*_ is the Fermi level. The *S* vs. *T* curve can be correlated via *∂*ln[*T*(*E*)]/*∂E*|_*E*=*E*_*F*__.  Figure 3(d) demonstrates that the normalized *S* (namely, each curve is upshifted to set the value of *S* at 6.50% to be 0 and thus the strain effect is removed) changes linearly as the temperature increases from 20°C to 60°C. The values of *S* for the slotted CNT (6,6) junction are always negative in the considered strain range (from 6.50% to 10.67%). This indicates the tail of LUMO dominates the contribution of transmission near *E*_*F*_ over that of HOMO does [14]. The bending deformation will arouse flexoelectric gating effect to this junction but not appreciably shift LUMO to the Fermi level and perform a limited influence on the term *∂*ln[*T*(*E*)]/*∂E*|_*E*=*E*_*F*__ [15]. For the bending stimulus, *ε*, varies from 6.50% to 10.67%, the changes in normalized *S* present little shifts by altering the ambient temperature, as characterized in Figure 3(d). The difference between each *S*–*T* curve under different strains is small. This implies mechanical deformation would have a small impact on temperature discrimination. Temperature perception is not affected by mechanical deformations. Thus, this slotted design enables reliable temperature monitoring under various applied bending strains.

It is crucial to distinguish the specific output signals from each stimulus that simultaneously act on the system. Here, the advance of the decoupled sensory performance to *ε* and *T* is due to the combination of two properties of cracked CNT junctions: bending leads to flexoelectricity, which driven Fano resonance with feedback *t*_*eff*_ reflecting the amplitude of the deformation, and the junction Seebeck coefficient provides the thermal signal that is slightly influenced by flexoelectric gating effect.

### 2.3. Single-Molecule Sensors

**Figure 4:**
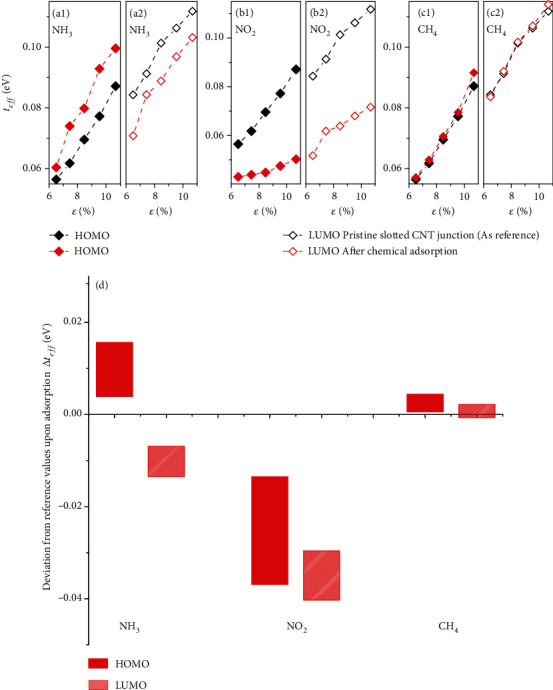
(a1)–(c2) Representative results of *t*_*eff*_ upon single molecular adsorption on the upper path measured at strain ranging from 6.50% to 10.67%, along with results from corresponding pristine junction (black data set). (d) A bar plot of changes in *t*_*eff*_ upon chemical adsorption in comparison with the pristine slotted junction for different levels of bending shown in (a1)–(c2).

In addition to bending and thermal sensory receptions above, we further demonstrate the expanded capabilities of this biomimetic slotted motif into effective high sensitivity chemical sensors. An illustration of a slotted CNT junction with response to three representative types of single molecular adsorbates on the upper path is shown in Figure 4. The width of each path is equivalent to four zigzag chains. For purpose of comparison, the samples include donor-type NH_3_, acceptor-type NO_2_, and inert CH_4_ molecules, along with the results of the corresponding pristine junction. Each molecule adsorbs at the hollow site, similar to the previous work [16, 17]. Signals are collected on the changes in HOMO and LUMO related *t*_*eff*_ at strain ranging from 6.50% to 10.67%. At the onset of single molecular adsorption on the upper path, the energy splitting between both paths (*t*_*eff*_) undergoes a bending-dependent geometric change, leading to a measurable difference with respect to those of pristine slotted CNT junction. The measured *t*_*eff*_ differences before and after molecular adsorption are clearly distinguishable for NH_3_ and NO_2_ in Figures 4(a1)–4(b2). Contrary to the chemically reactive NH_3_ and NO_2_, the changes of both HOMO and LUMO related *t*_*eff*_ for inert CH_4_ are small that lie within considered strain ranges in Figures 4(c1) and 4(c2). Calibration of *t*_*eff*_ can be performed with respect to that of the pristine CNT junction. By reading the deviations of *t*_*eff*_ to that in the pristine bendable slotted CNT junction (cf. △*t*_*eff*_ in Figure 4(d)), the corresponding adsorbed species can be readily determined.

It should be mentioned that the use of prestrained junction is necessary. Table S1 presents the △*t*_*eff*_ for NH_3_, NO_2_, and CH_4_ adsorption under two different conditions, normal flat and prebent samples. The signal of each molecule is determined through a modification of crosstalk between upper and lower paths, which enables conversion to the quantitatively measured changes in *t*_*eff*_. This is different from the previous work to characterize molecule based on Fano factor which reflects the weak interaction between the molecule and the carbon substrate [18, 19]. For the flat paths as substrate, only chemical gating is involved [20], and the determination of the accurate content for each target analyte would be restricted (only quantitative magnitude of △*t*_*eff*_). Whereas for prebent samples, the improvement is due to the combined effects of flexoelectric and chemical gating. It should be noted that the former gating stemming from strain effect would preserve the electron–hole symmetry of the slotted junction [21]. This has been experimentally confirmed on corrugated bilayer graphene system [22]. On the contrast, the chemical gating would break the electron–hole symmetry. This is in accordance with our analysis in Figures 1(c1) and 1(c2). As a result, this symmetry leads to the opposite evolution directions of the HOMO and the LUMO energies driven by increasing deformation (see Figures 2(a1) and 2(b1)), in contrast with those driven by enhancing the chemical gating (see Figures 2(a2) and 2(b2)). The associated trade-off between two types of gating could cause well-defined shifts in the predetermined *t*_*eff*_ by bending and promote the evaluation/assessment of these subtle variations in *t*_*eff*_ from both sign and magnitude. Furthermore, the predefined reference values of *t*_*eff*_ from prebent process can be used for filtering out the ambient noise to improve the reliability of the sensors against environmental variations. Therefore, this slotted platform with bendable deformation is a more rational strategy than the pristine flat one for the development of precise-resolution single molecular sensors.

To exploit the universality of this chemical sensor platform, we simulate the change of *t*_*eff*_ over eleven molecules by attaching each molecule to upper and lower paths as a function of bending strain. The results are summarized in Figure S1. The Fano factor provides crucial and distinct information for each species and confirms our sensor could successfully discriminate different types of molecules.

Generally, in order to acquire a significantly enhanced response of the molecular adsorbates, a large number of molecules are commonly required for chemical sensors. The electron transport process is typically off-resonant tunneling due to the molecular eigenvalues far away from the Fermi energy of the electrodes, and the sensor device does not have intrinsic chemical selectivity. In comparison with conventional CNT sensors [23], the architecture employed here offers an additional option (modified *t*_*eff*_) for delivering precise information of both chemically reactive and inert molecules which provides sufficient resolution to detect and identify individual chemical species. This is a critical need in countering threats in the detection and identification of toxic chemical at the lowest concentrations or response before concentrations reach dangerous levels. The ultimate limit on the sensitivity of chemical sensors would be at the single molecule level which is beneficial for the security issue. To gain ultrahigh sensitivity, confined sensing volume to the nanoscale would be promising [24, 25].

## 3. Discussions

By mimicking the fine structure from the spider leg (nanostructured slit), we designed a bioinspired multifunctional flexible device (Schematic 1) equipped within a single sensing unit, namely, a slotted CNT junction. For an initial proof of concept, we successfully demonstrated that such a junction could prevent cross-sensitivity in monitoring bending deformation and ambient temperature. Furthermore, this architecture enables detecting single molecular adsorption. This work provides a solid development platform for flexible electronics in monitoring sector. The proposed configurations are within the current experimental scope in three manners: CNT irradiated by energetic particles [26], selective chemical etching [27–31], and deriving carbon atomic chains in graphene [32, 33].

The simulation is implemented with modelling and ab initio transport calculations. The analytical models quantitatively reproduce the DFT and NEGF results, thereby underpinning the mechanism and providing guidelines for optimized designs. Our results indicated that different output signals extracted from the transmission spectrum under external stimuli could be clearly distinguished, which is important for decoupled multifunctional sensing performance. From an engineering standpoint, these issues in dealing with signal transduction and developing methods for converting mechanical, thermal, and chemical signatures to analyzable signals must be understood clearly before levels of integration in flexible electronics.

## 4. Methods

Structural relaxations were allowed until the force acting on each atom was less than 0.01 eV/Å. Then, transport calculations were performed based on DFT combined with the NEGF formalism [34]. Both calculations were performed using the QuantumATK package [35] within Perdew–Burke–Ernzerhof (PBE) density functional [36]. Van der Waals (vdW) corrections were included via the DFT-D2 method for physical adsorption of chemical molecules [37, 38]. For the transport calculations, three primitive layers of each electrode were included as buffer layers in the scattering region. The Brillouin zone was sampled with 1 × 1 × 150 Monkhorst meshes. We have explored various mechanical deformations, including bending and two types of compression (cf. Figure S2). We mainly focus on the bending operation, in which the quantum interference effect is significant.

## Figures and Tables

**Scheme 1:**
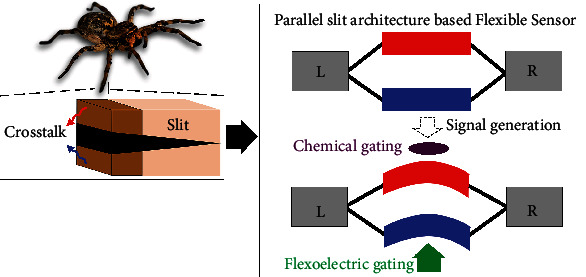
Schematic of how the spider's parallel slits organ served as the inspiration for developing a decoupled multimodal flexible sensor based on tunable Fano resonance.

## Data Availability

All data needed to evaluate the conclusions in the paper are presented in the paper and supplementary materials. And additional data are available from the corresponding authors upon reasonable request.

## Supplementary Materials

Supplementary Materials Figure S1: (a) bendable cracked CNT junction with molecular adsorption on upper path (U) and lower path (D). (b) The selectivity of single molecule was analyzed by monitoring the deviation of *t*_*eff*_ from the reference values upon adsorption on U or D (△*t*_*eff*_), where the reference values of *t*_*eff*_ are taken from the pristine slotted CNT junction with strain between 6.50% and 10.67%. Figure S2: (a1) atomic structures of the three types of the slotted CNT junction with deformation to accommodate the applied strain, designated as compression I, compression II, and bending. (a2) The relative energy differences of deformed CNT junctions to the flat one. (b1) Bending introduces the energy splitting of frontier orbitals between upper and lower paths of the slotted CNT junction attributed to flexoelectric gating. Evolution of this energy splitting as a function of strain deformation is shown in (b2). (c1) Compression I and compression II introduce energy gap (ELUMO-EHOMO) enlargement and shrinkage, respectively. The degeneracy of frontier orbitals for both paths is preserved in both deformations. Evolution of the gap as a function of strain deformation is shown (c2). “H” and “L” in Fig. b and c mean HOMO and LUMO, respectively, Table S1: comparison analysis of deviation from the reference values (△*t*_*eff*_) upon single molecule adsorption on upper path of the flat CNT junction and bendable CNT junction with strain between 6.50% and 10.67%. Only chemical gating is involved in the former set of data where the referenced values for flat CNT junction is 0.

## References

[1] Lee Y., Park J., Choe A., Cho S., Kim J., Ko H. (2020). Mimicking human and biological skins for multifunctional skin electronics. *Advanced Functional Materials*.

[2] Kim J., Lee M., Shim H. J. (2014). Stretchable silicon nanoribbon electronics for skin prosthesis. *Nature Communications*.

[3] Hua Q. L., Sun J. L., Liu H. T. (2018). Skin-inspired highly stretchable and conformable matrix networks for multifunctional sensing. *Nature Communications*.

[4] Ho D. H., Sun Q. J., Kim S. Y., Han J. T., Kim D. H., Cho J. H. (2016). Stretchable and multimodal all graphene electronic skin. *Advanced Materials*.

[5] Tien N. T., Jeon S., Kim D.-I. (2014). A flexible bimodal sensor array for simultaneous sensing of pressure and temperature. *Advanced Materials*.

[6] Zhang F. J., Zang Y. P., Huang D. Z., di C. A., Zhu D. B. (2015). Flexible and self-powered temperature-pressure dual-parameter sensors using microstructure-frame-supported organic thermoelectric materials. *Nature Communications*.

[7] You I., Mackanic D. G., Matsuhisa N. (2020). Artificial multimodal receptors based on ion relaxation dynamics. *Science*.

[8] Schaller R. R. (1997). Moore’s law: past, present and future. *IEEE Spectrum*.

[9] Fano U. (1961). Effects of configuration interaction on intensities and phase shifts. *Physical Review*.

[10] Lambert C. J. (2015). Basic concepts of quantum interference and electron transport in single-molecule electronics. *Chemical Society Reviews*.

[11] Kang D., Pikhitsa P. V., Choi Y. W. (2014). Ultrasensitive mechanical crack-based sensor inspired by the spider sensory system. *Nature*.

[12] Reuter M. G., Solomon G. C., Hansen T., Seideman T., Ratner M. A. (2011). Understanding and controlling crosstalk between parallel molecular wires. *Journal of Physical Chemistry Letters*.

[13] Lu H. Z., Lü R., Zhu B.-F. (2005). Tunable Fano effect in parallel-coupled double quantum dot system. *Physical Review B*.

[14] Reddy P., Jang S.-Y., Segalman R. A., Majumdar A. (2007). Thermoelectricity in molecular junctions. *Science*.

[15] Kim Y., Jeong W., Kim K., Lee W., Reddy P. (2014). Electrostatic control of thermoelectricity in molecular junctions. *Nature Nanotechnology*.

[16] Bai L., Zhou Z. (2007). Computational study of B- or N-doped single-walled carbon nanotubes as NH_3_ and NO_2_ sensors. *Carbon*.

[17] Thierfelder C., Witte M., Blankenburg S., Rauls E., Schmidt W. G. (2011). Methane adsorption on graphene from _first principles_ including dispersion interaction. *Surface Science*.

[18] Min S. K., Kim W. Y., Cho Y., Kim K. S. (2011). Fast DNA sequencing with a graphene-based nanochannel device. *Nature Nanotechnology*.

[19] Hong K., Kim W. Y. (2013). Fano-resonance-driven spin-valve effect using single-molecule magnets. *Angewandte Chemie-International Edition*.

[20] Zhang N., Lo W.-Y., Cai Z. X., Li L. W., Yu L. P. (2017). Molecular rectification tuned by through-space gating effect. *Nano Letters*.

[21] Jarillo-Herrero P., Sapmaz S., Dekker C., Kouwenhoven L. P., van der Zant H. S. J. (2004). Electron-hole symmetry in a semiconducting carbon nanotube quantum dot. *Nature*.

[22] Ho S.-C., Chang C.-H., Hsieh Y.-C. (2021). Hall effects in artificially corrugated bilayer graphene without breaking time-reversal symmetry. *Nature Electronics*.

[23] Schroeder V., Savagatrup S., He M., Lin S. B., Swager T. M. (2019). Carbon nanotube chemical sensors. *Chemical Reviews*.

[24] Wu Y. F., Tilley R. D., Gooding J. J. (2019). Challenges and solutions in developing ultrasensitive biosensors. *Journal of American Chemical Society*.

[25] Jones L. O., Mosquera M. A., Schatz G. C., Ratner M. A. (2019). Charge transport and thermoelectric properties of carbon sulfide nanobelts in single-molecule sensors. *Chemistry of Materials*.

[26] Krasheninnikov A. V., Banhart F. (2007). Engineering of nanostructured carbon materials with electron or ion beams. *Nature Materials*.

[27] Li J.-L., Kudin K. N., McAllister M. J., Prud’homme R. K., Aksay I. A., Car R. (2006). Oxygen-driven unzipping of graphitic materials. *Physical Review Letters*.

[28] Kosynkin D. V., Higginbotham A. L., Sinitskii A. (2009). Longitudinal unzipping of carbon nanotubes to form graphene nanoribbons. *Nature*.

[29] Kim G., Lee H.-J., Kwon Y.-K. (2013). Electronic properties of carbon nanotubes partially unzipped by oxygenation or fluorination. *Solid State Communications*.

[30] Lim J., Narayan Maiti U., Kim N.-Y. (2016). Dopant-specific unzipping of carbon nanotubes for intact crystalline graphene nanostructures. *Nature Communications*.

[31] Zhao Q. C., Yao F. R., Wang Z. Q. (2017). Real-time observation of carbon nanotube etching process using polarized optical microscope. *Advanced Materials*.

[32] Daaoub A., Lambert C. J., Sadeghi H. (2021). Genomics of carbon atomic chains. *Carbon*.

[33] Jin C. H., Lan H. P., Peng L. M., Suenaga K., Iijima S. (2009). Deriving carbon atomic chains from graphene. *Physical Review Letters*.

[34] Brandbyge M., Mozos J.-L., Ordejón P., Taylor J., Stokbro K. (2002). Density-functional method for nonequilibrium electron transport. *Physical Review B*.

[35] Smidstrup S., Markussen T., Vancraeyveld P. (2020). QuantumATK: an integrated platform of electronic and atomic-scale modelling tools. *Journal of Physics-Condensed Matter*.

[36] Perdew J. P., Burke K., Ernzerhof M. (1996). Generalized gradient approximation made simple. *Physical Review Letters*.

[37] Lee K., Murray É. D., Kong L. Z., Lundqvist B. I., Langreth D. C. (2010). Higher-accuracy van der Waals density functional. *Physical Review B*.

[38] Grimme S. (2006). Semiempirical GGA-type density functional constructed with a long-range dispersion correction. *Journal of Computational Chemistry*.

