# Supplementary material for: Flexoelectricity Driven Fano Resonance in Slotted Carbon Nanotubes for Decoupled Multifunctional Sensing
Source: Research (Wash D C). 2021 Dec 29;2021:9821905. doi: 10.34133/2021/9821905 (PMC8739842; doi:10.34133/2021/9821905)
Supplement: Supplementary Materials — Figure S1: (a) bendable cracked CNT junction with molecular adsorption on upper path (U) and lower path (D). (b) The selectivity of single molecule was analyzed by monitoring the deviation of teff from the reference values upon adsorption on U or D (△teff), where the reference values of teff are taken from the pristine slotted CNT junction with strain between 6.50% and 10.67%. Figure S2: (a1) atomic structures of the three types of the slotted CNT junction with deformation to accommodate the applied strain, designated as compression I, compression II, and bending. (a2) The relative energy differences of deformed CNT junctions to the flat one. (b1) Bending introduces the energy splitting of frontier orbitals between upper and lower paths of the slotted CNT junction attributed to flexoelectric gating. Evolution of this energy splitting as a function of strain deformation is shown in (b2). (c1) Compression I and compression II introduce energy gap (ELUMO-EHOMO) enlargement and shrinkage, respectively. The degeneracy of frontier orbitals for both paths is preserved in both deformations. Evolution of the gap as a function of strain deformation is shown (c2). “H” and “L” in Fig. b and c mean HOMO and LUMO, respectively, Table S1: comparison analysis of deviation from the reference values (△teff) upon single molecule adsorption on upper path of the flat CNT junction and bendable CNT junction with strain between 6.50% and 10.67%. Only chemical gating is involved in the former set of data where the referenced values for flat CNT junction is 0. [file 9821905.f1.pdf]

Supplementary Materials

Flexoelectricity Driven Fano Resonance in Slotted Carbon Nanotubes for Decoupled Multifunctional Sensing

Jinlong Ren[1], Yingchao Liu[1], Xingqiang Shi[3]*, Guangcun Shan[2,6]*, Mingming Tang[4], Chaocheng Kaun[5], Kunpeng Dou[1]*,

[1]College of Information Science and Engineering, Ocean University of China, Qingdao 266100, China.

[2] Institute of Precision Instrument and Quantum Sensing, School of Instrumentation Science and Opto-electronics Engineering, Beihang University, Beijing 100191, China.

[3] Key Laboratory of Optic-Electronic Information and Materials of Hebei Province, Institute of Life Science and Green Development, College of Physics Science and Technology, Hebei University, Baoding 071002, China.

[4]Research Institute of Unconventional Oil & Gas and Renewable Energy, China University of Petroleum, Qingdao, 266580, China.

[5]Research Center for Applied Sciences, Academia Sinica, Taipei 11529, China.

[6]Institute of Experimental Physics, Saarland University, 66123 Saarbrücken, Germany.

*Correspondence should be addressed to Xingqiang Shi;shixq20hbu@hbu.edu.cn, Guangcun Shan; gcshan@buaa.edu.cn,
and Kunpeng Dou; doukunpeng@ouc.edu.cn

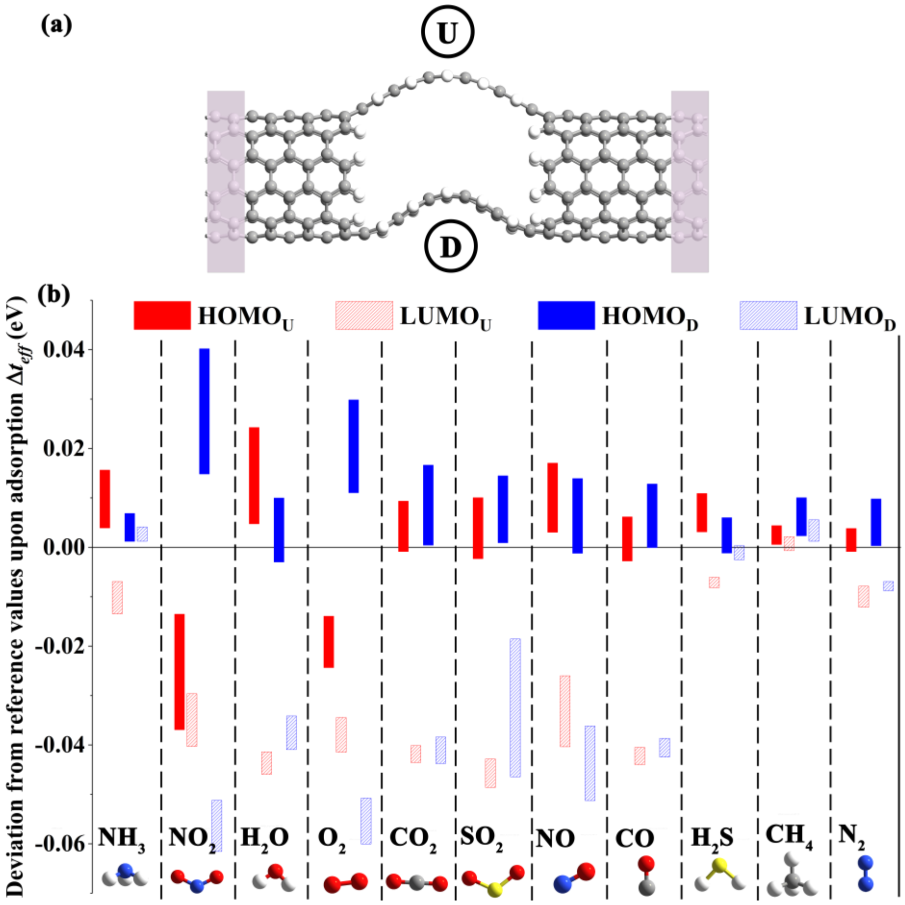

Figure S1. (a) Bendable cracked CNT junction with molecular adsorption on upper path (U) and lower path (D). (b) The selectivity of single molecule was analyzed by monitoring the deviation of t_{eff} from the reference values upon adsorption on U or D ($\triangle t_{eff}$), where the reference values of t_{eff} are taken from the pristine slotted CNT junction with strain between 6.50% and 10.67%.

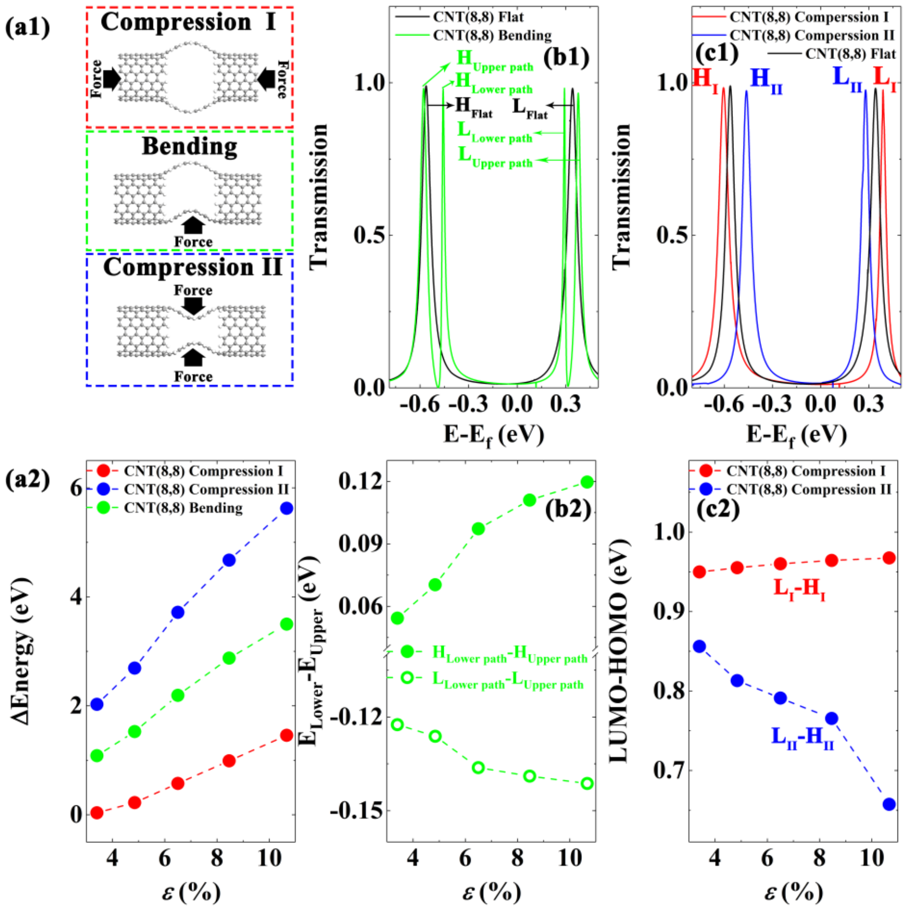

Figure S2. (a1) Atomic structures of the three types of the slotted CNT junction with deformation to accommodate the applied strain, designated as compression I, compression II and bending. (a2) The relative energy differences of deformed CNT junctions to the flat one. (b1) Bending introduces the energy splitting of frontier orbitals between upper and lower paths of the slotted CNT junction attributed to flexoelectric gating. Evolution of this energy splitting as a function of strain deformation is shown in (b2). (c1) Compression I and compression II introduce energy gap (E_{LUMO}-E_{HOMO}) enlargement and shrinkage, respectively. The degeneracy of frontier orbitals for both paths is preserved in both deformations. Evolution of the gap as a function of strain deformation is shown (c2). "H" and "L" in Fig. b and c mean HOMO and LUMO, respectively.

Table S1. Comparison analysis of deviation from the reference values ($\triangle t_{eff}$) upon single molecule adsorption on upper path of the flat CNT junction and bendable CNT junction with strain between 6.50% and 10.67%. Only chemical gating is involved in the former set of data where the referenced values for flat CNT junction is 0.

Adsorbate \ t_{eff} (eV)		Adsorbed on flat CNT (chemical gating)	Adsorbed on bending CNT (chemical gating + flexoelectric gating)
NH_3	HOMO	0.014	0.004 ~ 0.016
	LUMO	0.016	−0.014 ~ −0.007
NO_2	HOMO	0.035	−0.037 ~ −0.014
	LUMO	0.018	−0.040 ~ −0.030
CH_4	HOMO	0.002	0.001 ~ 0.004
	LUMO	0.007	−0.001 ~ 0.002

The model for a parallel-coupled double quantum dot (DQD) is described by the following Hamiltonian:

$$H = H_{leads} + H_{DD} + H_T \qquad \text{(S1)}$$

The H_{leads} stands for the noninteracting electron gas in the left (L) and right (R) leads,

$$H_{leads} = \sum_k \sum_{\alpha=L,R} \varepsilon_{k\alpha} c^+_{k\alpha} c_{k\alpha} \qquad \text{(S2)}$$

where $c^+_{k\alpha}$ ($c_{k\alpha}$) creates (annihilates) an electron in the lead α with energy $\varepsilon_{k\alpha}$.

The H_{DD} represents the electrons in weakly coupled quantum dots through tunneling,

$$H_{DD}=\sum_{i=1,2}\varepsilon_i d_i^+ d_i - t_c e^{i\theta} d_1^+ d_2 - t_c e^{-i\theta} d_2^+ d_1 \qquad \text{(S3)}$$

where d_i^+ (d_i) creates (annihilates) an electron in the dot i with energy ε_i. Besides, t_c and θ are the strength and the phase of interdot coupling, respectively. The energy ε_1 for dot 1 is different from ε_2 for dot 2 due to the applied electric field shown in Fig. 1a. There is no magnetic flux threading into the DQD-AB ring.

The tunneling between the leads and the dot is described by

$$H_T=\sum_k \sum_{\alpha=L,R} \sum_{i=1,2} V_{\alpha i} d_i^+ c_{k\alpha} + \text{H.c.} \qquad \text{(S4)}$$

where the tunneling matrix element $V_{\alpha i}$ is used to define the linewidth $\Gamma=\Gamma^L+\Gamma^R$ with the matrix element $\Gamma_{ij}^{\alpha}=\sum_k V_{\alpha i} V_{\alpha j}^* 2\pi\delta(\varepsilon-\varepsilon_{k\alpha})$. The linewidth matrices in the quantum dot representation is given by [13]

$$\Gamma^{\alpha}=\begin{pmatrix} \Gamma_1^{\alpha} & \sqrt{\Gamma_1^{\alpha}\Gamma_2^{\alpha}} \\ \sqrt{\Gamma_1^{\alpha}\Gamma_2^{\alpha}} & \Gamma_2^{\alpha} \end{pmatrix} = \begin{pmatrix} \Gamma_1 & \sqrt{\Gamma_1\Gamma_2} \\ \sqrt{\Gamma_1\Gamma_2} & \Gamma_2 \end{pmatrix} \qquad \text{(S5)}$$

where Γ_i^{α} is short for Γ_{ii}^{α} and for simplicity, symmetric coupling between two dots and the leads is considered with $\Gamma_i^L=\Gamma_i^R=\Gamma_i$.

To focus on the dot energy difference $\varepsilon_1-\varepsilon_2$ upon external electric field, it is instructive to map the parallel-coupled DQD to the DQD without the interdot coupling. In the latter case, the operator for a dot state can be expressed as

$$\begin{pmatrix} f_1 \\ f_2 \end{pmatrix} = \begin{pmatrix} \frac{1}{\sqrt{2}} & -\frac{1}{\sqrt{2}} \\ \frac{1}{\sqrt{2}} & \frac{1}{\sqrt{2}} \end{pmatrix} \begin{pmatrix} e^{-i\theta}\cos\beta & -\sin\beta \\ \sin\beta & e^{i\theta}\cos\beta \end{pmatrix} \begin{pmatrix} d_1 \\ d_2 \end{pmatrix} \qquad \text{(S6)}$$

where f_1 and f_2 are annihilation operators of states in two quantum dots in the right panel of Fig. 1a. The parameter β is defined as $\beta = 1/2\tan^{-1}\left[2t_c/\left(\varepsilon_1-\varepsilon_2\right)\right]$. Now the Hamiltonian for coupled dots in equation S3 is decoupled as

$$\tilde{H}_{DD} = \varepsilon_0\left(f_1^+ f_1 + f_2^+ f_2\right) + t_{eff}\left(f_1^+ f_2 + f_2^+ f_1\right) \quad \text{(S7)}$$

where the effective energy $\varepsilon_0 = \frac{\varepsilon_1+\varepsilon_2}{2}$ and effective coupling strength $t_{eff} = \sqrt{\frac{(\varepsilon_1-\varepsilon_2)^2}{4}+t_c^2}$. The new coupling matrix elements $V_{\alpha i}^*$ and linewidths Γ_i^* can be obtained by inserting equation (S6) into equation (S4). According to equation 20 in Ref. 13, the transmission spectrum for our system in the right panel of Fig. 1a can be solved as

$$T(E) = \frac{(\Gamma_1^*+\Gamma_2^*)^2\left[E-\varepsilon_0-\frac{2\sqrt{\Gamma_1^*\Gamma_2^*}}{\Gamma_1^*+\Gamma_2^*}t_{eff}\right]^2}{\left\{[E-(\varepsilon_0+t_{eff})]^2+\left[\frac{\left(\sqrt{\Gamma_1^*}-\sqrt{\Gamma_2^*}\right)^2}{2}\right]^2\right\}\left\{[E-(\varepsilon_0-t_{eff})]^2+\left[\frac{\left(\sqrt{\Gamma_1^*}+\sqrt{\Gamma_2^*}\right)^2}{2}\right]^2\right\}}$$

(S8)
